# Electroceutical fabric lowers zeta potential and eradicates coronavirus infectivity upon contact

**DOI:** 10.1038/s41598-021-00910-6

**Published:** 2021-11-05

**Authors:** Subhadip Ghatak, Dolly K. Khona, Abhishek Sen, Kaixiang Huang, Gargi Jagdale, Kanhaiya Singh, Vinoj Gopalakrishnan, Kenneth G. Cornetta, Sashwati Roy, Savita Khanna, Lane A. Baker, Chandan K. Sen

**Affiliations:** 1 Indiana Center for Regenerative Medicine and Engineering at Indiana University School of Medicine, Indianapolis, IN 46202 USA; 2 Department of Chemistry, Indiana University, Bloomington, IN 47405 USA; 3 Department of Medical and Molecular Genetics at Indiana University School of Medicine, Indianapolis, IN USA

**Keywords:** Microbiology, Antimicrobials, Virology

## Abstract

Coronavirus with intact infectivity attached to PPE surfaces pose significant threat to the spread of COVID-19. We tested the hypothesis that an electroceutical fabric, generating weak potential difference of 0.5 V, disrupts the infectivity of coronavirus upon contact by destabilizing the electrokinetic properties of the virion. Porcine respiratory coronavirus AR310 particles (10^5^) were placed in direct contact with the fabric for 1 or 5 min. Following one minute of contact, zeta potential of the porcine coronavirus was significantly lowered indicating destabilization of its electrokinetic properties. Size-distribution plot showed appearance of aggregation of the virus. Testing of the cytopathic effects of the virus showed eradication of infectivity as quantitatively assessed by PI-calcein and MTT cell viability tests. This work provides the rationale to consider the studied electroceutical fabric, or other materials with comparable property, as material of choice for the development of PPE in the fight against COVID-19.

The basic reproductive number of an infection, denoted as R_o_, gauges the number of susceptible individual(s) that an infectious host can spread their disease^1^. Respiratory infections are known to spread through direct routes of aerosolization such as sneezing, coughing and other contact gestures^2,3^. In addition, indirect modes of transmission play a significant role in determining R_o_. SARS-CoV-2 remains viable for an extended period of time. The CDC reported presence of SARS-CoV-2 RNA on various surfaces on the Diamond Princess ship 17 days after all symptomatic and asymptomatic COVID-19 passengers had vacated^4,5^. In a laboratory-based experimental study, SARS-CoV-2 remained viable for at least three hours in aerosols and up to 72 h on fomites such as stainless steel^6^. Use of personal protective equipment (PPE) is essential to safeguard healthcare providers against COVID-19^7^. However, use of these PPE itself poses significant threat as doffing of contaminated PPE carrying viable viral particles is likely to infect the person and potentially spread infection^8^. Although CDC has recommended strict doffing procedures to reduce risk of nosocomial infections, contaminated PPE poses an imminent serious risk for healthcare professionals^9–12^.

Infectivity of a viral particle is dependent on its stability. Multiple biophysical factors determine the stability of coronaviruses^13^. For instance, nonspecific electrostatic interactions influence capsid assembly of enveloped RNA viruses in which positively charged capsid proteins package the negatively charged RNA. In positive-sense single-stranded RNA viruses, such as the coronaviruses, this thermodynamically spontaneous assembly is mediated by arginine rich motifs. A large number of ss-RNA-viruses follow a general law of packaging, based on electrostatic forces without an explicit dependence on the sequence specificity^14^. The overall positive charge of the capsid limits viral genome length^15,16^. However, coronaviruses express an exoribonuclease associated with nonstructural protein 14 which allows them to inherit longer genomes when compared to other RNA viruses^17^. Destabilization of coronavirus outside the host system is therefore of paramount importance in abating the spread of infection. We have co-developed a wireless electroceutical fabric for use as wound care dressing^18–22^. This dressing, upon contact with bodily fluids or other aqueous wetting media, generates weak electric field which is effective in managing biofilm infection and improving wound healing^18,20,21,23^. The dressing is currently FDA cleared and in clinical trial (NCT04079998). In this work we sought to investigate the effectiveness of the said electroceutical fabric for its ability to curb coronavirus infectivity.

## Results

### Characterization of the electroceutical fabric

**Figure 1:**
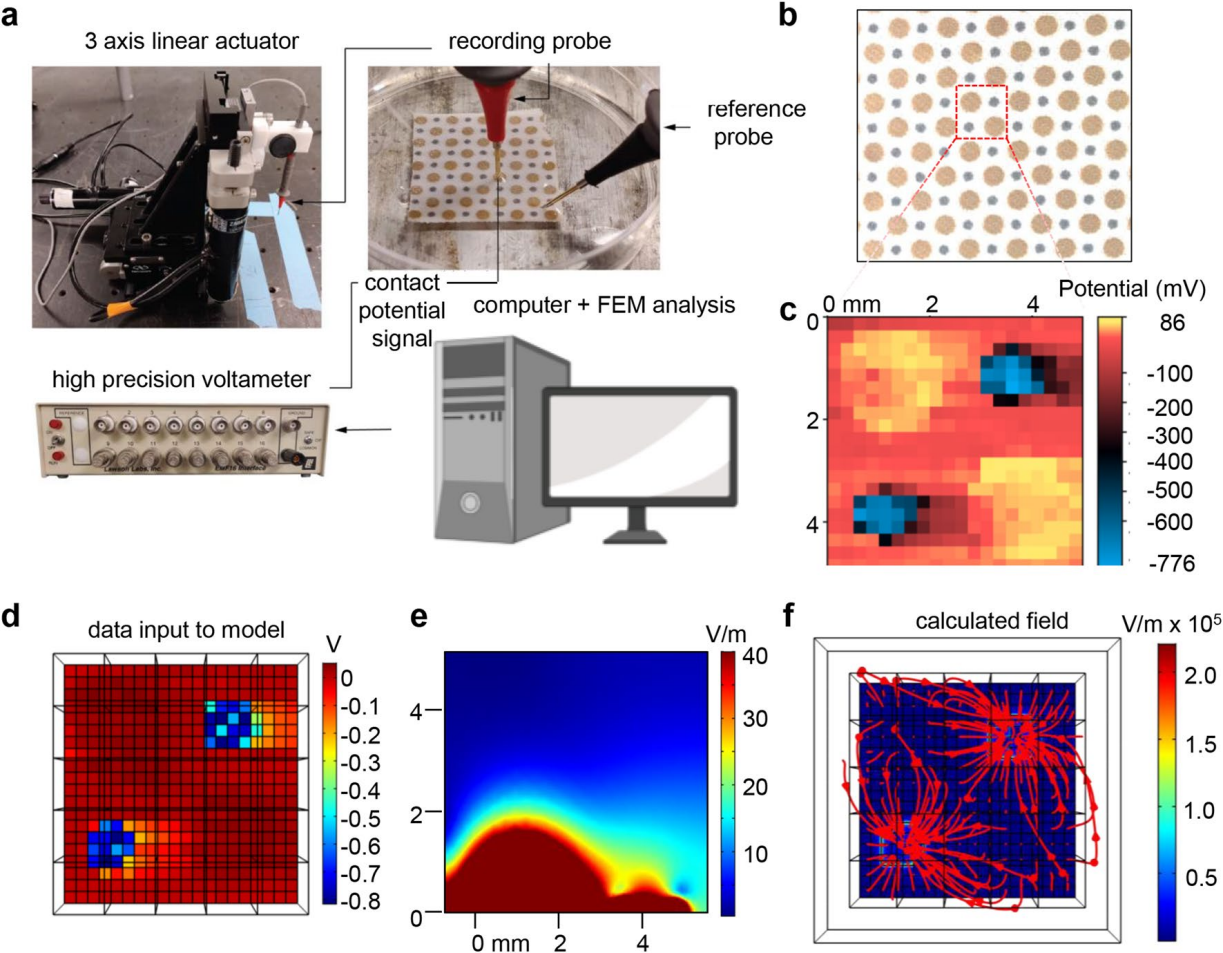
Contact potential mapping on electroceutical fabrics. (**a**) Schematic of the contact potential mapping measurement. A gold-coated probe tip mounted on a 3-axis linear actuator system was raster scanned over the sample area and recorded the contact potential at each probed position with reference to the unprinted fabrics. The potential was read by a high precision voltameter, followed by data analysis and finite element modeling (FEM) simulation. Figure created with BioRender.com (https://app.biorender.com/). (**b**) Photomicrograph of the electroceutical fabric. (**c**) Two-dimensional surface potential map of a region similar to the dashed square in (**b**) in presence of EMEM media. The averaged potentials of Zn and Ag were measured as -650 mV and + 60 mV, respectively. (**d**) Top view of the three-dimensional FEM model constructed from (**c**). (**e**) Calculated E-field map from FEM simulations in the perpendicular direction from the fabric surface. A slice of the field values was selected that crossed both Ag and Zn electrode. (**f**) Top view of the simulated vector field image indicating the distribution and magnitude of the electric field over the fabric surface.

**Figure 2:**
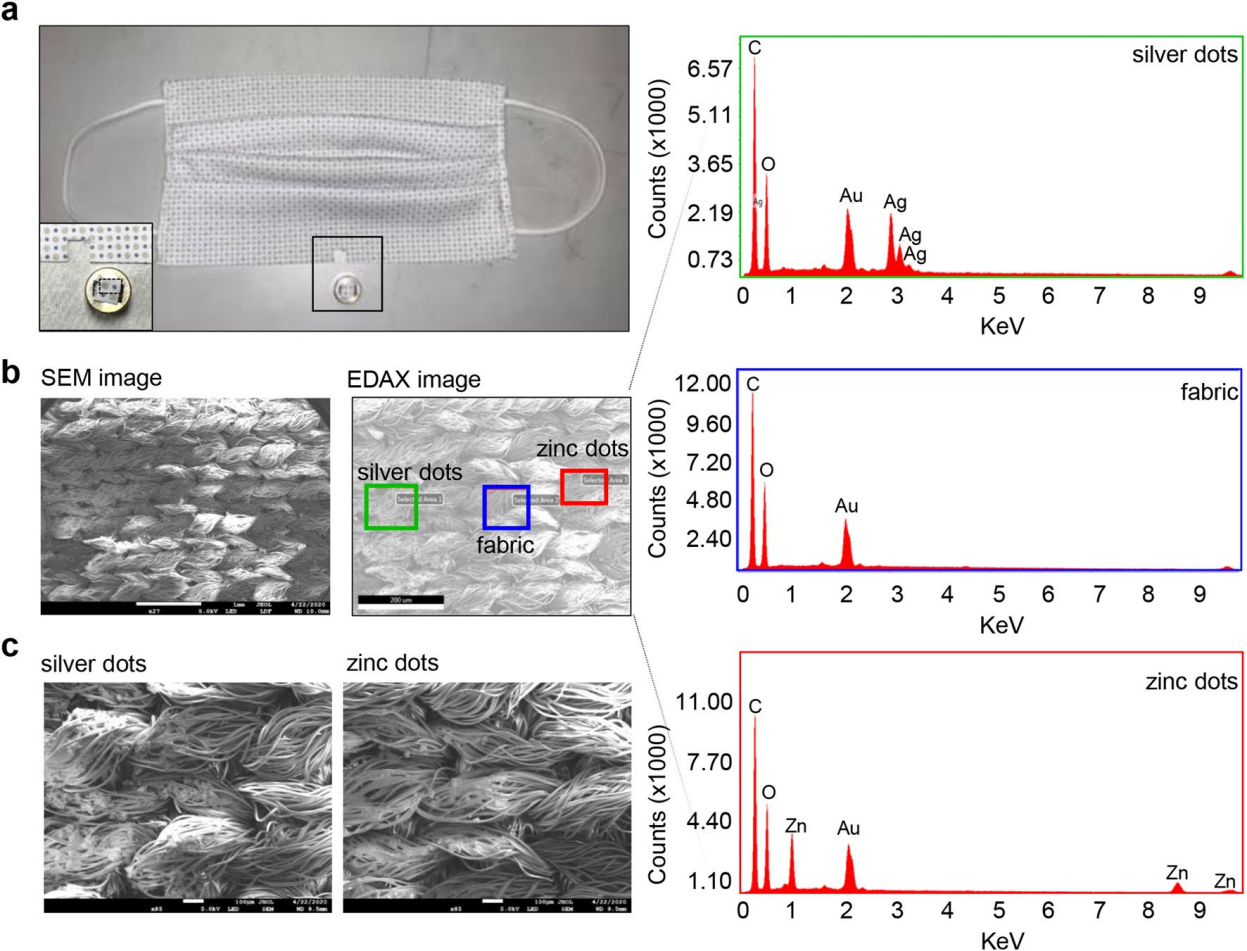
The prototype face mask utilizing stretchable electroceutical textile. (**a**) Photomicrograph of the region cut from the face mask and used for SEM and EDX analysis. (**b** and **c**) SEM and EDX analysis for silver and zinc deposition on the textile. Regions of mask used for EDX analysis have been marked and annotated in the respective figure panel.

**Figure 3:**
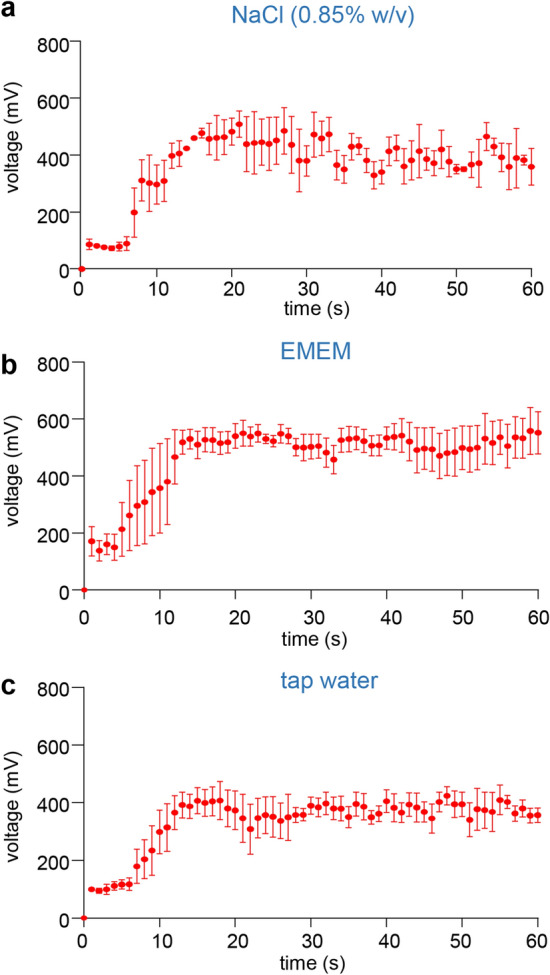
The electroceutical fabric. Voltage generated by electroceutical fabric was measured using the Amprobe multimeter in three different aqueous wetting solutions: (**a**) NaCl (0.85% w/v); (**b**) Incomplete EMEM, and (**c**) Tap water. DC voltage was measured as shown. Probes were placed adjacent Ag and Zn dots and at 0 s, 100 µl of the respective wetting solution were added to the electroceutical fabric. Three independent readings (each lasting for 1 min) were recorded for all three solutions and graphs were plotted with mean of these readings, showing the activation kinetics of the electroceutical fabric in response to these wetting solutions. Data are mean ± SEM.

The electroceutical fabric tested is made up of polyester fabric printed with alternating circular regions of Ag and Zn dots (Fig. 1b). The Ag dots (ø 2 mm) and Zn dots (ø 1 mm) were printed on the fabric in proximity of about 1 mm to each other. The fabric was first characterized by measurements of the surface contact potential of a two-dimensional area covering 2 Ag dots and 2 Zn dots, with reference to the potential of the unprinted polyester fabric (Fig. 1a, Supplementary Fig. 1). The electroceutical fabric only generate measurable electric potentials when moist such as EMEM media. Two-dimensional contact surface potential map demonstrates Ag spots as positive while Zn spots were highly negative (Fig. 1c). The average potential of Ag spots and Zn spots were calculated to be + 60 mV and − 650 mV, respectively suggesting that printed metal patterns on fabric can generate stable electrical potential. Finite element modeling (FEM) simulations (Fig. 1d) showed that electric fields project to a significant degree above the fabric with field strength dropping to 5 V/m around 3 mm above the fabric surface (Fig. 1e), with the majority of the electric field accumulated at the Zn dots (Fig. 1f). Ag and Zn were spotted on another textile which was also appropriate for the preparation of stretchable facemasks (Fig. 2a). Scanning electron microscopy (SEM) displayed the deposition of Ag particles and Zn on the fibers of the polyester fabric of stretchable facemasks (Fig. 2b). SEM of the fabric used for such mask showed a different weaving pattern (Fig. 2b). EDX microanalysis revealed the presence of Ag and Zn on the electroceutical fabric (f_e_) and absence in the sham polyester fabric (f_s_) (Fig. 2c and Supplementary Fig. 2). The only peak that was present other than C and O was that of Au used for coating the fabrics for SEM imaging (Supplementary Fig. 2a). Proximity of Ag and Zn on polyester fabric forms a redox couple and is capable of driving electrochemistry when wet in an aqueous ionized environment including any body fluid (Fig. 1). Three ionized aqueous media were used to test potential difference between adjacent Ag and Zn deposits. NaCl solution (0.85% w/v), cell culture medium and tap water (of practical value to end users of PPE) were tested at room temperature. The potential difference between the two electrodes rapidly increased and achieved a steady state after the first 15 s (Fig. 3). The potential difference between adjacent Ag and Zn dots was also sustained in presence of different body fluids (Supplementary Fig. 3).

### Physical characterization of the coronavirus

SEM (150,000×) revealed the morphological features of the porcine corona virus (CoV) particle (Supplementary Fig. 4a). Following spotting on the silicon wafer, the purified virus was fixed and subsequently dehydrated. A thin (2–5 nm) layer of carbon was sputtered on the sample to make the specimen conductive. The size of the virus ranged between 75–125 nm. Nanoparticle tracking analysis (NTA) revealed poly-dispersed peak (Supplementary Fig. 4b). The electrokinetic property, as represented by the zeta(ζ) potential, of the viral particles is a parameter that determines adsorption and stability of the particle in any given dispersant medium. For practical purposes, viral particles are expected to be suspended in water droplets either aerosolized or resting on a surface. The average ζ potential of four different preparation of CoV was determined to be − 25.675 mV (Supplementary Fig. 4c). All four-preparation demonstrated comparable ζ potential distribution and phase shift (Supplementary Fig. 4d–e). The average electrophoretic mobility distribution was determined to be − 2μmcm/Vs (Supplementary Fig. 4f).

### Electroceutical fabric attenuated the ζ potential of coronavirus upon contact

**Figure 4:**
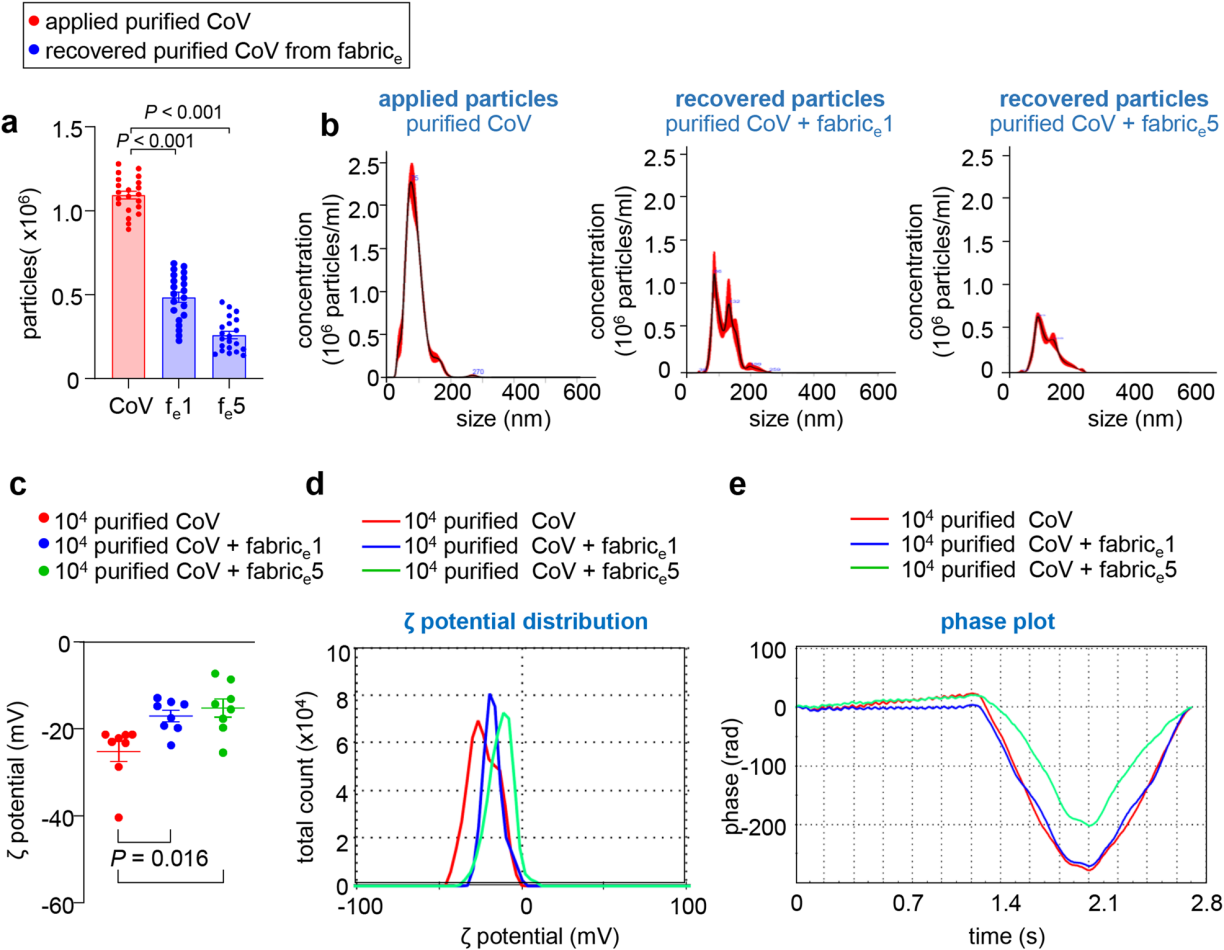
Zeta potential and nanosight tracking analysis of the purified coronavirus following contact with and retrieval from the electroceutical fabric. (**a**) Absolute quantification of viral particles recovered from the fabric after treatment with f_e_. A two-fold and four-fold reduction in the recovered viral number was observed after 1- and 5-min treatment, respectively. (**b**) Viral particle number was quantified using NTA. One hundred microliters of the purified CoV were spotted on 1.5 cm discs of electroceutical fabric and recovered using incomplete EMEM, after 1 or 5 min of contact with the fabric. (**c**–**e)** Changes in viral zeta potential and affiliated parameters after contact with the fabric for 1 min or 5 min. Data are mean ± SEM.

Quantification of the viral particles after spotting on f_e_ yielded 44.29% and 23.73% recovery from the fabric when exposed for 1 min or 5 min, respectively (Fig. 4a). Nanoparticle tracking analysis demonstrated that unlike the purified CoV that showed a single peak around 75 nm, the recovered CoV showed additional peaks suggesting aggregation of the viral particles upon contact with the fabric (Fig. 4b). Analysis of ζ potential showed significant graded attenuation of this electrokinetic property upon contact with the f_e_ (Fig. 4c). Such lowering of average ζ potential of CoV, applied and recovered from f_e_, has been plotted (Fig. 4d). Unlike 1 min exposure to the f_e_, 5 min exposure showed an appreciable difference in the phase plot of the viral particles (Fig. 4e).

### Loss of coronavirus infectivity upon contact with electroceutical fabric

**Figure 5:**
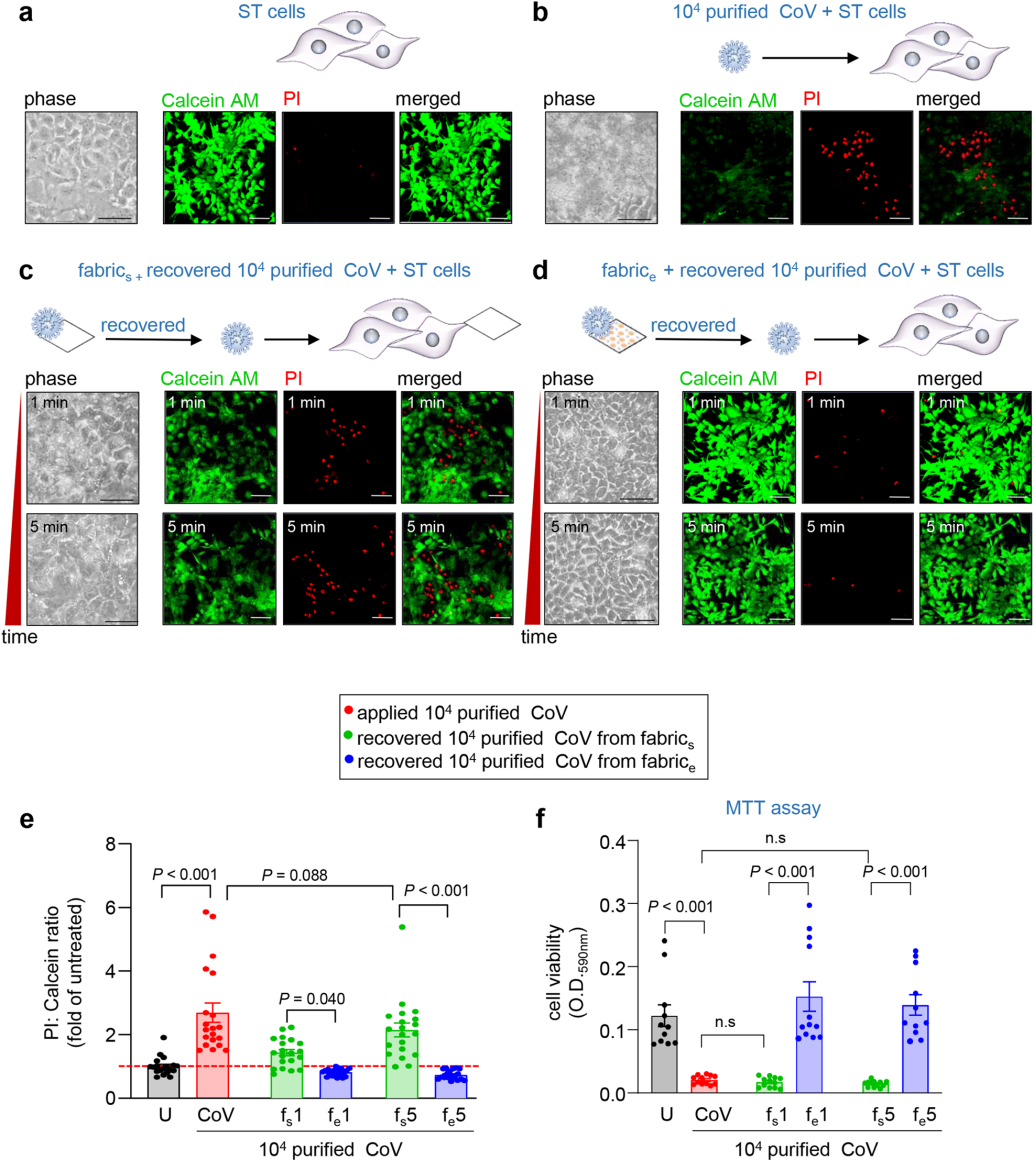
Eradication of respiratory coronavirus infectivity upon contact with the electroceutical fabric. ST cells were infected with respiratory CoV (4 × 10^4^ viruses). In other test sets, the virus (10^5^) was brought in contact with the fabric for either 1 min or 5 min. After 7 days of infection, cells were observed for cytopathic effects (CPE, rounding and sloughing) by phase contrast microscopy. Host cell viability was objectively quantified using dual staining with Calcein AM (green, viable) and PI (red, non-viable). (**a**) uninfected ST cells (u); (**b**) ST cells infected with virus (CoV, 4 × 10^4^); (**c**) ST cells infected with viruses pre-exposed to the sham fabric (f_s_1 and f_s_5); and (**d**) ST cells infected with viruses pre-exposed to the electroceutical fabric for 1 or 5 min, respectively (f_e_1 and f_e_5). ST cells infected with untreated virus or f_s_-contacted virus showed distinct signs of CPE and loss of cell viability. Cells infected virus which were subjected to contact with the electroceutical fabric for 1 (f_e_1) or 5 (f_e_5) minute did not display any further loss of cell viability above and beyond the basal level of cell death expected at that phase of the life-cycle of the cell. (**e**) Quantitative plotting of changes in cell viability as determined by PI/calcein expressed as fold-change over the basal cell death level expected as part of standard cell culture process. (**f**) Changes in cell viability as determined by the MTT (3-(4,5-dimethylthiazol-2-yl)-2,5-diphenyltetrazolium bromide) assay. Scale bars in images represent 100 µm. Phase contrast images were captured at 40× magnification. Corresponding zoom out images showing a larger field of view were taken at 20× and presented as Supplementary Fig. 4. Data are mean ± SEM. Figure created using MS PowerPoint.

To assess changes in the infectivity of CoV following contact with the electroceutical fabric, a cytopathic assay was employed. Infected cells were monitored for appearance of cytopathic effects (CPE; cell rounding and sloughing) until post-infection day 7. Overt CPE was observed on day 7 in response to CoV infection (Fig. 5b; Supplementary Fig. 5aii). Comparable CPE was noted in response to treatment of cells with CoV recovered from sham control fabric f_s_ (Fig. 5c; Supplementary Fig. 5aiii–iv). In contrast, CoV recovered from f_e_ did not cause any CPE indicating loss of its infectivity (Fig. 5d; Supplementary Fig. 5av–vi). Cells treated with f_e_-recovered CoV particles appeared as healthy as the uninfected cells (Fig. 5a; Supplementary Fig. 5ai). Objective assessment of cell viability was performed using calcein/PI fluorescence assay. Only live cells with intracellular esterase activity hydrolyze the acetoxymethyl ester in non-fluorescent Calcein AM converting it into green fluorescent Calcein. Dead cells or cells with damaged or compromised cell membranes include PI stain, which is otherwise impermeant to live cells. Fold-change increase in PI/Calcein signal as shown indicates loss of cell viability in response to infection. Infection of cells with CoV caused marked loss of cell viability (Fig. 5b). Such cytopathic effect of CoV was completely absent once the virus was exposed to f_e_ (Fig. 5d, e). The sham fabric did not afford such protection (Fig. 5c, e). The cytopathic effects of CoV and the protective effects of f_e_ (versus f_s_) was corroborated by the standard MTT assay commonly used for testing cell viability (Fig. 5f). The cytopathic effects of CoV were primarily due to increase generation of H_2_O_2_ in the ST cells that were abrogated when the virus particles were exposed to f_e_ (Supplementary Fig. 5b). The protective effects of f_e_ were further supported by cellular respirometry assay that showed increased basal respiration, maximum respiration and ATP production in cells exposed to CoV recovered from f_e_ **(**Supplementary Fig. 5c–e)_._

## Discussion

Previous work from our laboratory has established the effectiveness of electroceutical principles as an alternative to pharmacological approaches in managing planktonic microbial pathogens and complex polymicrobial biofilms^18,20,21,23,24^. Viruses are known to rely on electrostatic interactions for optimal virion assembly and attachment^13,25^. For instance, structural proteins in coronaviruses, negatively charged amino acid residues in the nucleocapsid facilitates assembly with the membrane protein^26^. Additionally, the coronavirus envelope protein is known to generate ion conductive pores across membranes which are voltage dependent^27^. Leveraging these viral characteristics to achieve viral inactivation remains largely unexplored and has been attempted in this work.

Electroceuticals have generated renewed interest in the health care industry^28^. The fabric tested in this work consists of only silver and zinc dots on polyester fabric that forms a redox couple^18–23^. Zeta potential of a particle determines its electrostatic interactions in particle dispersions and, as such, is an important determinant of the stability of viral particles^29^. Contact of CoV with the electroceutical fabric studied rapidly lowered the zeta potential demonstrating a direct effect of the fabric on the electrokinetic properties of the viral particle. Any change of zeta potential towards zero is viewed as increase in electrical instability of the particle. The observation that in contact with the electroceutical fabric eliminates infectivity of the virus leads to the hypothesis that the observed lowering of zeta potential may have caused defects in the structural integrity of the virus. Study of changes in the capsid-RNA structure following exposure to the weak electric field generated by the fabric is thus warranted.

CoV is a nanoparticle. Nanoparticle tracking analysis determines the hydrodynamic diameter of the analyte by applying the Stokes–Einstein equation after measuring the Brownian motion of individual nanoparticle^30^. NTA was utilized to estimate absolute viral particle number and size distribution in not only pure CoV but also in CoV recovered from the fabric. Observed changes in particle number and size distribution support the aforementioned hypothesis that exposure to the weak electric field causes damaging structural alterations to the virions. Cells in culture routinely display a small fraction of dead or dying cells. Cytopathic effects of viral infection are tested to examine whether exposure to the infectious particle adds to the basal cell death burden of the culture. Long-term observations, i.e. days versus hours, ensure the recording of the eventual fate of the affected cells. Reporting of short-term data alone, while sometimes may be encouraging with respect to effect of the intervention, may simply reflect results representing postponement of death from the insult and not a true rescue. In CPE studies of this work, cell rounding, and sloughing were evident in day 4 post-infection. During this time, cells treated with virus pre-exposed to the electroceutical fabric closely resembled cells that were unchallenged by exposure to the virus. In standard cell culture, the growth medium is changed every other day to wash off floating dead cells and to replenish nutrition. Under conditions of infection by virus, such frequent change of cell culture medium is not made. Cells grow in the same spent media until day 7 post-infection. Maintenance of cells without any change in culture media for seven days is expected to marginally increase basal cell death burden as shown.

Textiles evaluated for use in PPE such as masks are subject to specific FDA 510(k) requirements expecting stringent viral filtration tests to demonstrate 99.9% reduction of 1.1–3.3 × 10^4^ plaque forming units of standard phiX174 bacteriophage. The phiX174 is widely used as a model organism because of it being a standardized test. However, it is important to note that unlike SAR-CoV-2 which is a RNA virus, phiX174 bacteriophage is a DNA virus with numerous contrasting physical, chemical as well as biological properties. Furthermore, this bacteriophage is much smaller in size than SAR-CoV-2. The non-enveloped icosahedral morphology of phiX174 are aerosolized with a mean particle size of 3.0 ± 0.3 µm^31^. This is in direct contrast with the coronaviruses that cause diseases in animals and humans which are ~ 100 nm in diameter and are aerosolized as respiratory droplets with sizes > 5 µm^32,33^. Importantly, phiX174 cannot infect mammalian cells. It infects and forms visible plaques on a lawn of *Escherichia coli* (Migula) Castellani and Chalmers strains. In the context of COVID-19 pandemic, this work studies the coronavirus and tests cytopathic effects on mammalian cells. Testing methods such as AATCC TM100 recommends a textile contact time of 24 h for both enveloped and non-enveloped viruses. We report results on contact time that is much shorter and more relevant to PPE usage in the context of COVID-19.

This work presents first evidence demonstrating that the physical characteristic features of CoV may be exploited to render it non-infective following exposure to weak electric field generating electroceutical fabric. The observation that lentiviral infectivity is also eliminated following contact with the electroceutical fabric contributes to the rigor of our central finding. Lowering of zeta potential of the CoV particles following exposure to the electroceutical fabric constitutes direct evidence supporting the contention that electrokinetic stability of the viral particle is weakened. Additional studies are necessary to characterize specific structural changes in response to exposure to the electroceutical fabric, and to connect such changes to loss of infectivity. In the meanwhile, this work provides evidence supporting the rationale to consider the studied electroceutical fabric, or other materials with similar property, as material of choice for the development of PPE in the fight against COVID-19.

## Methods

### Electroceutical fabric

An FDA cleared wireless electroceutical dressing was used as a source of weak electric field for the current study and is referred to as electroceutical fabric (f_e_). This fabric, co-developed by our laboratory^18–22^, has been commercialized by Vomaris Inc. (Phoenix, AZ) was provided to us by the manufacturer. It is made of polyester fabric printed with alternating circular dots of Ag and Zn metals (ø2 mm and 1 mm, respectively), generating electric fields. A polyester fabric without any metal deposition (hence unable to generate electric field) was used as an experimental control and is referred to as sham fabric (f_s_).

### Contact potential mapping for dressings

Two-dimensional maps of the surface potential of the electroceutical dressing were collected by the modified version of a method reported previously^34^. Briefly, a gold-coated probe tip (100 µm diameter) was mounted on a microprobing system consisting of miniature linear actuators (Zaber Technologies Inc., Vancouver, Canada) with independent control in the XYZ dimensions. The dressings were moistened by EMEM media and mounted on the sample stage. With the microprobing system, the probe tip raster scanned to contact the sample surface for potential measurement at each pixel. The scan area was 5 × 5 mm in 20 × 20 pixels covering 2 Ag dots, 2 Zn dots and empty dressing. The potential at the probe tip was referenced to the empty dressing, as measured by a high precision and high input impedance voltmeter (EMF 16, Lawson Lab Inc., Malvern, PA, USA) at each pixel. The collected data were plotted and processed by Gwyddion^35^. The finite element model was constructed and analyzed by COMSOL Multiphysics 5.4.

### Collection of body fluids

Human body fluids such as blood, saliva and sweat were collected from healthy adult volunteers at Indiana University Health Comprehensive Wound Center (IUH-CWC). Human wound fluid samples were obtained from chronic wound patients undergoing Negative Pressure Wound Therapy (NPWT) dressing (sponges) by lavaging the wound dressing with saline solution. All human studies were approved by Indiana University’s Institutional Review Board. Declaration of Helsinki protocols was followed, and patients gave their written informed consent.

### Viruses and cell lines

Porcine respiratory coronavirus (ATCC® VR-2384™) AR310 and its host porcine cell line - ST (ATCC® CRL-1746™) were procured from ATCC.

### Cell culture

ST (ATCC® CRL-1746™) were cultured and maintained in complete Eagle's Minimum Essential Medium (EMEM, ATCC® 30-2003™) at 37 °C and humidified 5% CO_2_ in air atmosphere. All culture media were made complete by addition of Fetal Bovine Serum (FBS, final concentration 10%, Sigma-Aldrich, F2442) and antibiotic–antimycotic solution (final concentration 1X; 15240-062, Life Technologies).

### Respiratory coronavirus infection and propagation

ST cells were cultured in complete EMEM till they attained a confluency of 80–90% followed by washing monolayers with 5 ml of 1X PBS. Coronavirus stock (ATCC VR-2384™; USDA permit 141794) aliquot was diluted with 3 ml of incomplete culture medium (without FBS and antibiotic–antimycotic solution) to attain a Multiplicity of Infection (MOI) of 1. This diluted viral stock was added to the washed monolayer and incubated at 37 °C, humidified 5% CO_2_ in air atmosphere. Flasks were rocked gently for 2 min at intervals of 30 min, to re-distribute viral inoculum. After 2 h infection, viral adsorption was terminated by adding 10 ml of complete culture medium to the monolayer.

### Coronavirus purification

Coronavirus purification was performed as described previously^36^. In brief, culture medium from flasks with infected cells was harvested at 10,000×g for 20 min at 4 °C. Viral precipitation from this supernatant (12 ml) was done by addition of polyethylene glycol and NaCl. PEG precipitated proteins and virions were pelleted at 10,000×g for 30 min at 4 °C and the pellet was dissolved in 100 µl of ice-cold 1X HEPES-saline buffer (10 mM HEPES – Sigma H7523 + 0.9% w/v NaCl, pH 6.7). Dissolved pellet was then loaded on a discontinuous sucrose gradient (10–20–30%, 800 µl each; in 1X HEPES-saline) and subjected to ultracentrifugation at 100,000×g for 90 min at 4 °C.

### Nanoparticle tracking analysis

Viruses were diluted in EMEM or 18.2 MΩ water. Mean particle diameter and concentration of viral particles were analyzed by NanoSight NS300 with a 532 nm laser and SCMOS camera (Malvern) as previously described^37–39^. Standard 100 nm latex spheres were run at 1000:1 dilution in milliQ to test optimal instrument performance. Data were analyzed using NTA 3.0 software (Malvern Instruments).

### Zeta potential analysis

Zeta (ζ) potential measurement of viral particles was determined by Zetasizer (Nano-Z, Malvern Instruments Ltd., UK) as described previously^37–39^. All samples were dispersed in double-distilled water and tested in volume-weighted size distribution mode in folded capillary cells (Fisher Scientific NC0491866). An average of three readings (60 s) were recorded.

### Scanning electron microscopy

Viral particles were suspended in ddH2O with 2.5% glutaraldehyde or other buffer and dropped onto clean silica wafers. After drying, samples were desiccated in a vacuum chamber for at least 12 h before analysis. Images were obtained after carbon sputter coating using a field emission scanning electron microscope (JEOL 7800F, JEOL Japan) at a beam energy of 5 or 10 kV. For the SEM images of the fabric, gold sputter coating was used.

### Energy dispersive X-ray (EDX) microanalysis

For elemental detection, the EDX microanalysis associated with SEM was used^40^. The x-ray emissions at different wavelengths were measured using a photon-energy-sensitive detector. The EDX detector system performed a simultaneous display of all mid-energy (1–20 keV) x-rays collected during any individual analysis period.

### Coronavirus infectivity

ST (coronavirus) cells were seeded at densities of 10,000 /well and 1000/well in 24-well and 96-well cell culture plates, respectively. Seeded plates were incubated at 37 °C, 5% CO_2_ humidified incubator for 18 h. One hundred microliter (10^5^ particles) of aqueous suspension of viruses (10^6^/ml of AR310) were placed on 1.5 cm diameter discs of f_e_ and f_s_ at room temperature and time was allowed for the droplet to be fully absorbed into the respective fabric. As soon as that was achieved, a timer was started for either 1 min or 5 min of contact time followed by recovery of the particles. For 10^5^ AR310 viral particles that were placed in direct contact with the fabric, 2.5–4 × 10^4^ particles were recovered from the fabric. Serum free medium (100 µl) was used to rinse each fabric for recovering viral particles from the fabric. NTA was performed, as above, to estimate viral recovery efficiency. Recovered VR-2384 viruses were diluted with serum free medium and used to infect ST cells at MOI of 10 (10^5^ viruses). Parallel sets of cells infected with untreated viruses (at the same MOI as that of treated viruses) were used as positive control while uninfected or non-transduced host cells were accounted as negative control.

### Cell viability staining by calcein AM and propidium iodide

Viability of ST cells, infected as above, was assessed by dual staining with Calcein AM and Propidium iodide (PI)^41,42^. Media from wells with ST cells (uninfected or infected with untreated or fabric-contacted viruses) was washed briefly with 1 ml of 1X PBS (per well) for 1 min, followed by addition of 250 µl of freshly-prepared staining solution in 1X PBS (Calcein AM; final concentration 1 µg/ml, Catalog no: C1430, Invitrogen™) and PI (Sigma-Aldrich). Cells were incubated under dark conditions at 37 °C for 15 min and then observed under a Confocal Laser Scanning Microscope using a 10× objective. The ratio of PI:calcein signal was normalized with the average PI intensity of untreated cells to obtain fold-change compared to non-viable cells (basal cell death) in untreated cells.

### Cell viability assessment by MTT assay

Cell viability of ST cells infected as above was assayed using the MTT (3-(4,5-Dimethylthiazol-2-yl)-2,5-diphenyltetrazolium bromide) assay as per manufacturer’s protocol (MTT assay kit, Catalog No.: ab211091, Abcam).

### Respirometry assay

Mitochondrial stress was measured with Sea Horse XFe96 Analyzer. Cells (5 × 10^3^) were seeded, in EMEM supplemented with 10% FBS and 1% A/A, in a 96-well Sea Horse XFe96 cell culture microplate. Once adhered, the cells were exposed to viral particles, before and recovery from electroceutical fabric. Post virus exposure, cells were washed thrice with PBS and re-suspended in Sea Horse XF base medium (DMEM base with 25 mM glucose, 4 mM glutamine and 1 mM pyruvate, pH 7.4,). Thereafter, keratinocytes were incubated in a non-CO_2_ incubator for 1 h at 37 °C. Meanwhile, mitochondrial regulators oligomycin, CCCP and antimycin and rotenone was added to the Sea Horse XFe96 cartridge. Then, the cartridge containing mitochondrial regulators was mounted on the top of the Seahorse XFe96 utility plate containing XF calibrant for hydrating the analyzer sensors. The duo was, thereafter, inserted into the Seahorse XFe96 Analyzer. After calibrating the sensors, the machine ejected the utility plate and cell culture plate was inserted. The respirometry assay was, then, run at 37 °C wherein oligomycin, CCCP, and antimycin and rotenone were sequentially administered to keratinocytes. Once the run is complete, alterations in cellular oxygen consumption rates were measured.

### Cytosolic H_2_O_2_ measurement

H_2_O_2_ abundance was measured using pHyPer-cyto. Cells (7.5 × 10^3^ cells/well) seeded in Nunc Lab-Tek II 8-well chamber slides were transfected with highly sensitive genetically encoded H_2_O_2_ probe, pHyPer-cyto (500 ng/µl). The transfected cells were exposed to viral particles, before and recovery from electroceutical fabric. Post virus exposure, cell medium was decanted off from chamber slides, washed thrice with PBS, fixed in IC fixation buffer, stained with nucleic acid stain (DAPI) (0.1 ng/µl), and mounted with VectaMount aqueous mounting medium and visualized for H_2_O_2_ generation through confocal microscope (Zeiss LSM 880). Signal quantitation was done using ZEN 3.0 software.

### Statistical analysis

GraphPad Prism (GraphPad Software) v8.0 was used for statistical analyses. Statistical analysis between multiple groups were performed using one-way analysis of variance with the post-hoc Sidak multiple comparison test. Statistical analysis between two groups was performed using unpaired Student’s two-sided t tests. *P* < 0.05 was considered statistically significant. Significance levels and exact *P* values are indicated in all relevant figures. Data were normally distributed. Data for independent experiments were presented as means ± SEM unless otherwise stated. Individual data points are plotted reflecting n (8–19) for each experiment.

## Supplementary Information

Supplementary Information.

## References

[1] van den Driessche P (2017). Reproduction numbers of infectious disease models. Infect. Dis. Model.

[2] Jayaweera M, Perera H, Gunawardana B, Manatunge J (2020). Transmission of COVID-19 virus by droplets and aerosols: A critical review on the unresolved dichotomy. Environ. Res..

[3] Dhand R, Li J (2020). Coughs and sneezes: Their role in transmission of respiratory viral infections, including SARS-CoV-2. Am. J. Respir. Crit. Care Med..

[4] Oliver, D. Coronavirus genetic material stayed on surfaces for up to 17 days on Diamond Princess cruise, CDC says. March 30, 2020. USA Today. https://www.usatoday.com/story/travel/cruises/2020/03/24/coronavirus-diamond-princess-cabin-surfaces-contaminated-cdc-report/2905924001/

[5] Mojica, A. CDC examination of COVID-19 on cruise ships found COVID-19 RNA on surfaces for 17 days. March 25, 2020. *Fox17*. https://fox17.com/news/local/cdc-examination-of-covid-19-on-cruise-ships-found-virus-on-surfaces-for-17-days

[6] van Doremalen N (2020). Aerosol and surface stability of SARS-CoV-2 as compared with SARS-CoV-1. N. Engl. J. Med..

[7] Holland M, Zaloga DJ, Friderici CS (2020). COVID-19 personal protective equipment (PPE) for the emergency physician. Vis. J. Emerg. Med..

[8] Wang D (2020). Clinical characteristics of 138 hospitalized patients with 2019 novel coronavirus-infected pneumonia in Wuhan, China. JAMA.

[9] CDC. Use personal protective equipment (PPE) when caring for patients with confirmed or suspected COVID-19. https://www.cdc.gov/coronavirus/2019-ncov/downloads/A_FS_HCP_COVID19_PPE.pdf

[10] Young, R. knowing how to remove PPE is a matter of life and death, one ER doctor says. April 14, 2020. *Wbur.* https://www.wbur.org/hereandnow/2020/04/14/emergency-room-doctor-coronavirus

[11] Cook TM (2020). Personal protective equipment during the coronavirus disease (COVID) 2019 pandemic: A narrative review. Anaesthesia.

[12] Gordon C, Thompson A (2020). Use of personal protective equipment during the COVID-19 pandemic. Br. J. Nurs..

[13] Perlmutter JD, Hagan MF (2015). Mechanisms of virus assembly. Annu. Rev. Phys. Chem..

[14] Forrey C, Muthukumar M (2009). Electrostatics of capsid-induced viral RNA organization. J. Chem. Phys..

[15] Belyi VA, Muthukumar M (2006). Electrostatic origin of the genome packing in viruses. Proc. Natl. Acad. Sci. U. S. A..

[16] Hu T, Zhang R, Shkovskii BI (2008). Electrostatic theory of viral self-assembly. Phys. A.

[17] Minskaia E (2006). Discovery of an RNA virus 3'->5' exoribonuclease that is critically involved in coronavirus RNA synthesis. Proc. Natl. Acad. Sci. U. S. A..

[18] Banerjee J (2015). Silver-zinc redox-coupled electroceutical wound dressing disrupts bacterial biofilm. PLoS ONE.

[19] Banerjee J (2014). Improvement of human keratinocyte migration by a redox active bioelectric dressing. PLoS ONE.

[20] Barki KG (2019). Electric field based dressing disrupts mixed-species bacterial biofilm infection and restores functional wound healing. Ann. Surg..

[21] Ghatak PD (2015). A wireless electroceutical dressing lowers cost of negative pressure wound therapy. Adv. Wound Care (New Rochelle).

[22] Vilkhu R (2018). Power generation for wearable electronics: Designing electrochemical storage on fabrics. IEEE Access.

[23] Khona DK (2021). Ketoconazole resistant *Candida albicans* is sensitive to a wireless electroceutical wound care dressing. Bioelectrochemistry.

[24] Roy S (2019). Disposable patterned electroceutical dressing (PED-10) is safe for treatment of open clinical chronic wounds. Adv. Wound Care.

[25] Liu YC, Grusovin J, Adams TE (2018). Electrostatic interactions between hendra virus matrix proteins are required for efficient virus-like-particle assembly. J. Virol..

[26] Kuo L, Masters PS (2002). Genetic evidence for a structural interaction between the carboxy termini of the membrane and nucleocapsid proteins of mouse hepatitis virus. J. Virol..

[27] Verdia-Baguena C (2012). Coronavirus E protein forms ion channels with functionally and structurally-involved membrane lipids. Virology.

[28] Reardon S (2014). Electroceuticals spark interest. Nature.

[29] Ohshima H (2009). Theory of electrostatics and electrokinetics of soft particles. Sci. Technol. Adv. Mater..

[30] Vestad B (2017). Size and concentration analyses of extracellular vesicles by nanoparticle tracking analysis: A variation study. J. Extracell Vesicles.

[31] Rengasamy S, Shaffer R, Williams B, Smit S (2017). A comparison of facemask and respirator filtration test methods. J. Occup. Environ. Hyg..

[32] Shiu EYC, Leung NHL, Cowling BJ (2019). Controversy around airborne versus droplet transmission of respiratory viruses: Implication for infection prevention. Curr. Opin. Infect. Dis..

[33] Tellier R, Li Y, Cowling BJ, Tang JW (2019). Recognition of aerosol transmission of infectious agents: A commentary. BMC Infect. Dis..

[34] Park SS, Kim H, Makin IR, Skiba JB, Izadjoo MJ (2015). Measurement of microelectric potentials in a bioelectrically-active wound care device in the presence of bacteria. J. Wound Care.

[35] Necas D, Klapetek P (2012). Gwyddion: an open-source software for SPM data analysis. Cent. Eur. J. Phys..

[36] Maier HJ, Bickerton E, Britton P (2015). Coronaviruses methods and protocols preface. Methods Mol. Biol..

[37] Ghatak S (2016). AntihypoxamiR functionalized gramicidin lipid nanoparticles rescue against ischemic memory improving cutaneous wound healing. Nanomedicine.

[38] Li J (2018). Topical lyophilized targeted lipid nanoparticles in the restoration of skin barrier function following burn wound. Mol. Ther..

[39] Zhou X (2020). Exosome-mediated crosstalk between keratinocytes and macrophages in cutaneous wound healing. ACS Nano.

[40] Scimeca M, Bischetti S, Lamsira HK, Bonfiglio R, Bonanno E (2018). Energy dispersive X-ray (EDX) microanalysis: A powerful tool in biomedical research and diagnosis. Eur. J. Histochem..

[41] Khanna S (2013). Loss of miR-29b following acute ischemic stroke contributes to neural cell death and infarct size. J. Cereb. Blood Flow Metab..

[42] Khanna S (2003). Molecular basis of vitamin E action: Tocotrienol modulates 12-lipoxygenase, a key mediator of glutamate-induced neurodegeneration. J. Biol. Chem..

