# Supplementary material for: Electroceutical fabric lowers zeta potential and eradicates coronavirus infectivity upon contact
Source: Sci Rep. 2021 Nov 5;11:21723. doi: 10.1038/s41598-021-00910-6 (PMC8571396; doi:10.1038/s41598-021-00910-6)
Supplement: Supplementary file 1 — Supplementary Information. [file 41598_2021_910_MOESM1_ESM.pdf]

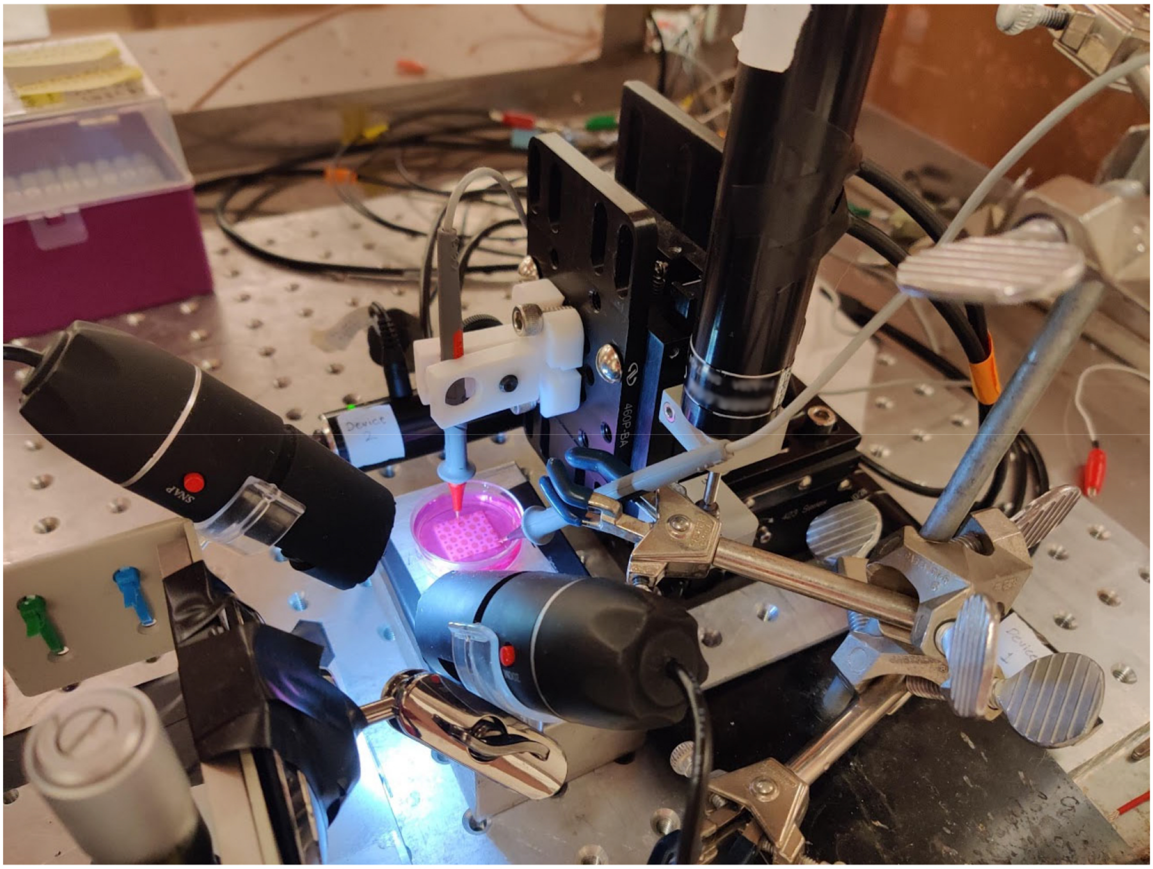

Supplementary Figure 1 | Photo showing the instrument setup of contact potential measurement on electroceutical fabrics.

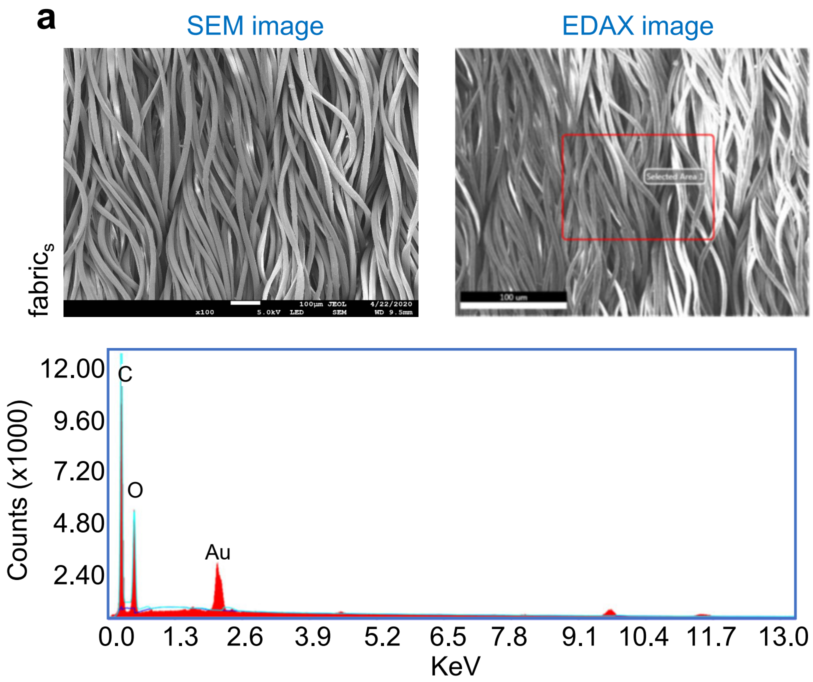

Supplementary Figure 2 | Energy Dispersive X-Ray microanalysis (EDX) of sham fabric

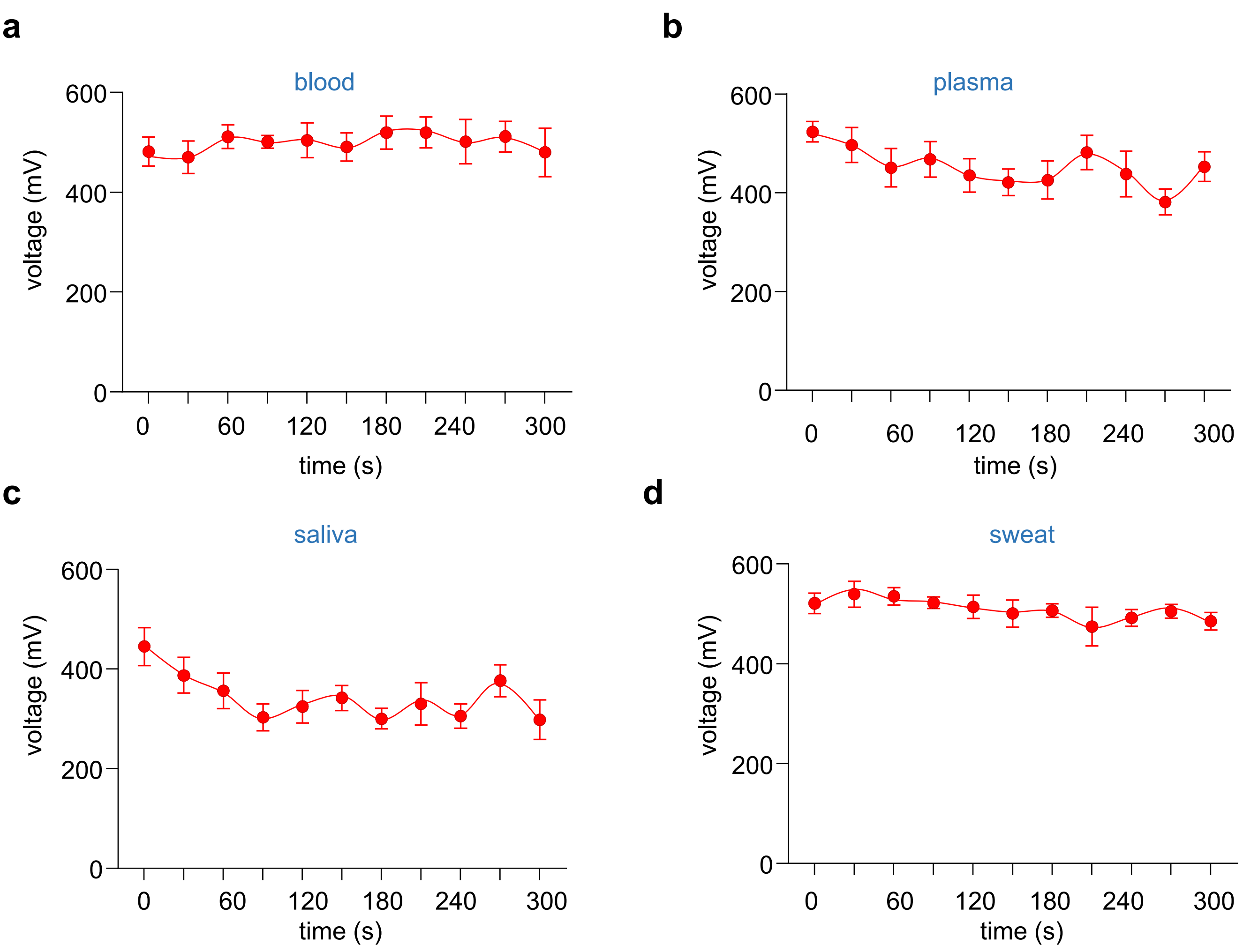

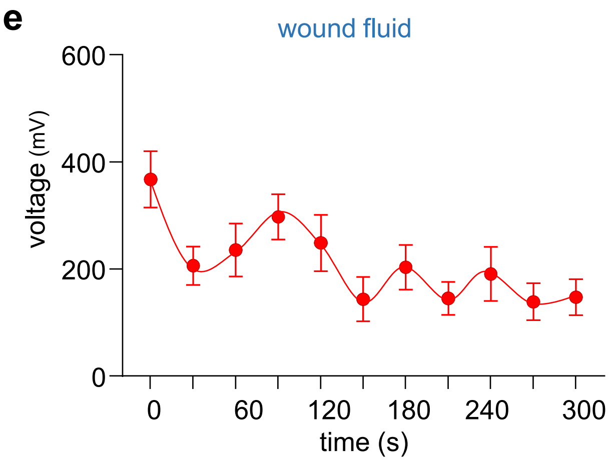

Supplementary Figure 3 | Voltage generated by electroceutical fabric in presence of body fluid. The voltage generated was measured using the Amprobe multimeter in different body fluids: **a**, blood (n=10); **b**, plasma (n=9); **c**, saliva (n=10); **d**, sweat (n=5) and **e**, wound fluids (n=7). DC voltage was measured as shown. Probes were placed adjacent Ag and Zn dots and at 0s, 100-200 µl of the respective fluids were added to the electroceutical fabric.

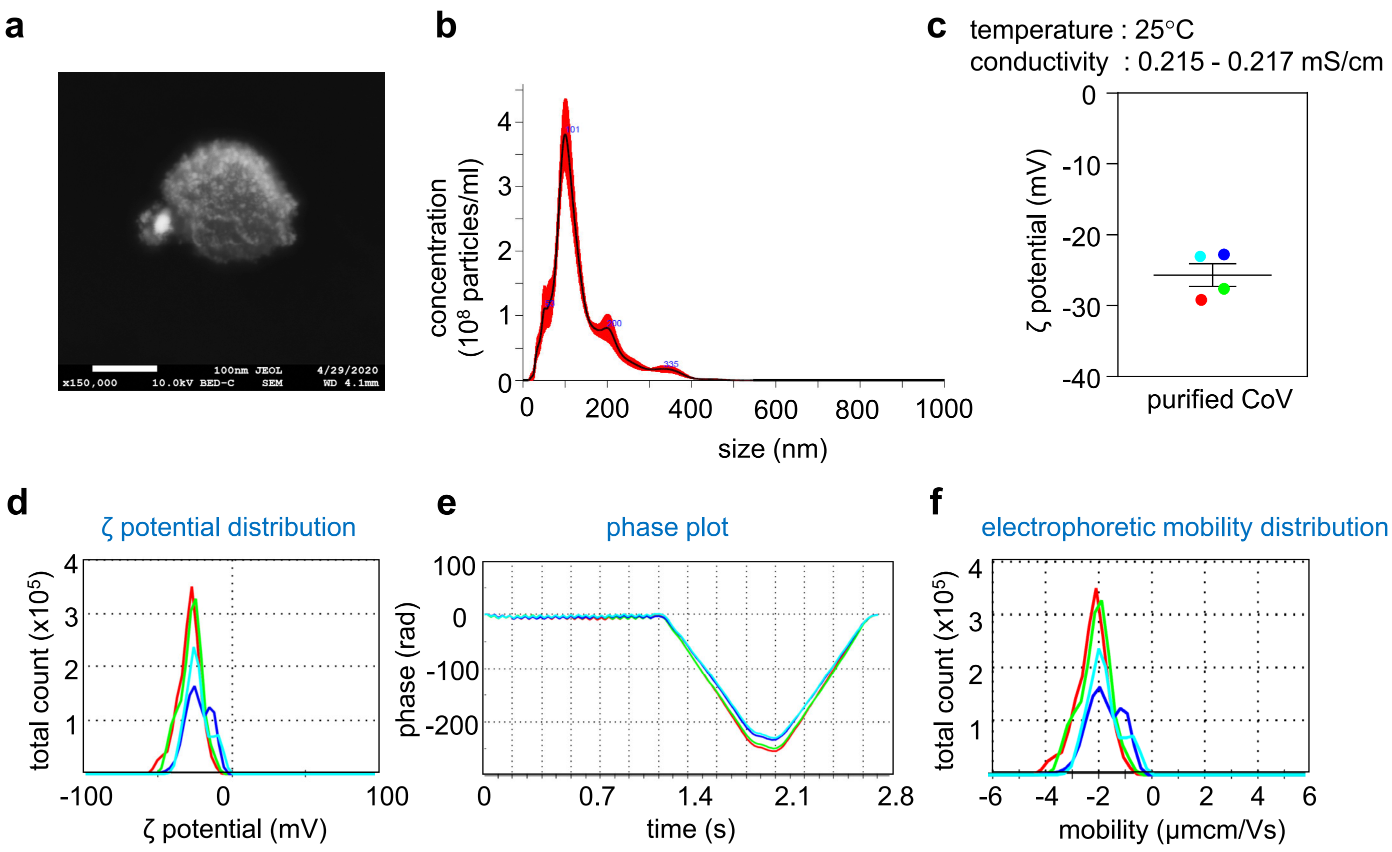

Supplementary Figure 4 | Physical characterization of the purified coronavirus. (**a**) SEM image of purified respiratory CoV. **b,** Viral particle number in the purified respiratory CoV sample was quantified using NTA. An estimated yield of 4×10^8 viruses with one major peak corresponding to 100 nm size, was obtained from the adopted viral purification protocol. (**c – f**) Zeta potential readouts of four independent (mean ± SEM shown) purified coronavirus sample preparation and the different attributes of analyses are depicted. **c,** Individual zeta potential values of purified respiratory CoV suspended in 18.2 MΩ water. **d,** Zeta potential distribution within individual reads. **e,** Individual phase plots determined by applying alternating voltage and reaching minimum between 1.75 – 2.00 s. **f,** Electrokinetic observation obtained by measuring mobility of the virus in response to an external electrical field.

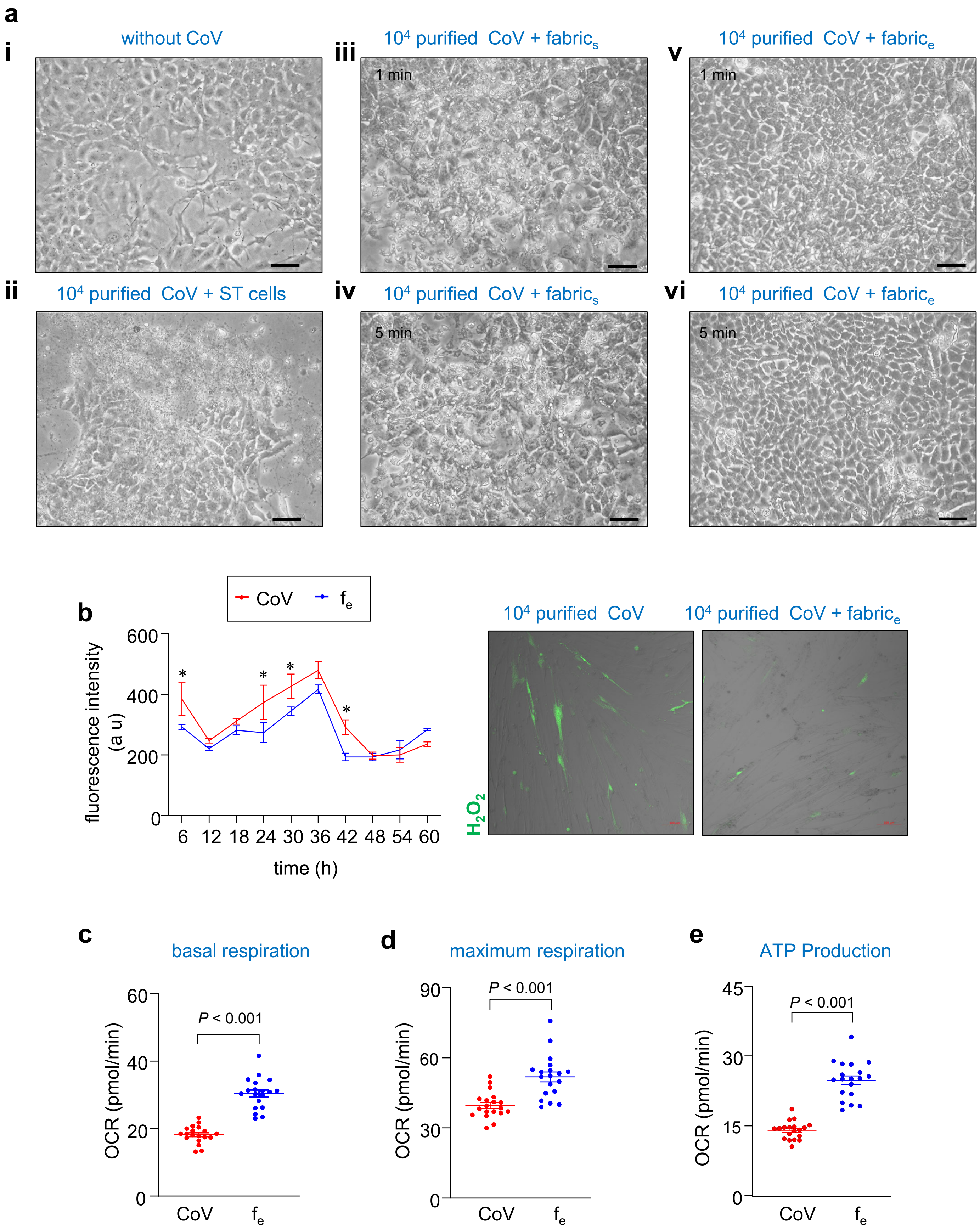

a
i
without CoV
ii
10^4 purified CoV + ST cells
iii
10^4 purified CoV + fabric$_s$
1 min
iv
10^4 purified CoV + fabric$_s$
5 min
v
10^4 purified CoV + fabric$_e$
1 min
vi
10^4 purified CoV + fabric$_e$
5 min
b
CoV
f$_e$
fluorescence intensity (a u)
time (h)
0
200
400
600
6 12 18 24 30 36 42 48 54 60
*
10^4 purified CoV
10^4 purified CoV + fabric$_e$
H_2O_2
c
basal respiration
d
maximum respiration
e
ATP Production
OCR (pmol/min)
P < 0.001
CoV
f$_e$
0
20
40
60
0
30
60
90
0
15
30
45

Supplementary Figure 5 | a, Phase contrast microscopy images of ST cells, uninfected or infected with CoV (either treated with fs or fe for 1 min or 5 min). These images are taken at 20X magnification displaying a larger field of view. Zoom-in images (40X) shown Figure 5. Scale bars, 100 µm. **b,** CoV exposure results in reactive oxygen species generation. * P <0.01. (n=3) **c-e,** The basal respiration, maximum respiration and ATP production in cells treated exposed with CoV.
